# First detection of white spot syndrome virus (WSSV) in the mud shrimp *Austinogebia edulis* in Taiwan

**DOI:** 10.1038/s41598-019-54837-0

**Published:** 2019-12-09

**Authors:** Fei Zhu, Wen-Hung Twan, Li-Chun Tseng, Shao-Hung Peng, Jiang-Shiou Hwang

**Affiliations:** 1 Zhejiang Provincial Engineering Laboratory for Animal Health Inspection and Internet Technology, College of Animal Science and Technology, Zhejiang Agriculture and Forestry University, Hangzhou, China; 2 Department of Life Science, National Taitung University, Taitung, Taiwan; 3 Institute of Marine Biology, National Taiwan Ocean University, Keelung, Taiwan; 4 Center of Excellence for the Oceans, National Taiwan Ocean University, Keelung, Taiwan; 5 Center for Research in Water Science and Technology, National Cheng Kung University, Tainan, Taiwan

**Keywords:** Biological techniques, Biotechnology

## Abstract

The white spot syndrome virus (WSSV) causes mass mortalities in the aquaculture of shrimps worldwide. The mud shrimp *Austinogebia edulis* (Ngoc-Ho & Chan, 1992) is an economically important sea food item occurring along the west coast of Taiwan. While the population of *A. edulis* began to decrease with some fluctuations in the last decade, the current study aims to discover the causes for such sporadic population decline. This study explores the effects of microbial pathogens and innate immunity on the populations of *A. edulis*. Here, we report firstly about WSSV infection of *A. edulis* from the coastal zone of western Taiwan which is one of the possible causes of population decrease of *A. edulis* in Shengang. However, WSSV infection is not the only reason for its population decrease because a similar infection rate of WSSV was found in Wangong. Population changes may be related to both environmental pollution stress and WSSV. Both factors likely caused a massive reduction of hemocytes and an abnormal increase of phenoloxidase and superoxide dismutase activity, which were spectrophotometrically measured. Since there is no effective way to treat WSSV infection, improving the coastal environment appears the most effective way to increase the population size of feral shrimps.

## Introduction

In Taiwan the white spot syndrome virus (WSSV) was first discovered in 1992. Ever since it has resulted in mass mortalities and substantial production losses in the aquaculture of shrimps in several Taiwan counties^1–4^. The genome of WSSV is approximately 300 kb in size and indicates its allocation to the genus *Whispovirus*, the only genus of the family Nimaviridae^5,6^.

WSSV can lead to a total mortality of shrimps in aquaculture within one week^7^. WSSV can infect several crustacean species in natural, man-made, and experimental settings^8–11^. Evidence of histopathological manifestations in target organs is one of the criteria used in the diagnosis of WSSV infection^7,12^. During WSSV infection, gills and hepatopancreas organs are severely damaged. Pathological features such as organ necrosis, encapsulation, and nodule formation in the hepatopancreas were described, providing initial indications of the WSSV disease^13^.

**Figure 1:**
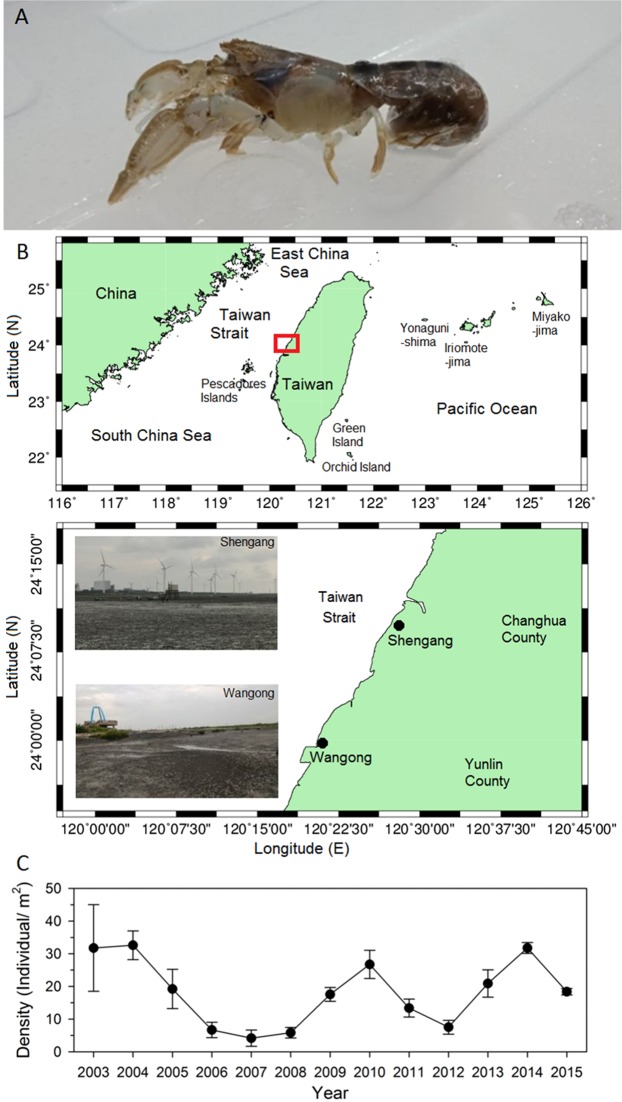
Mud shrimp *Austinogebia edulis* (**A**), map of the sampling area indicating the location of the collection sites Shengang and Wangong in western Taiwan (**B**), and the population of *A. edulis* in Shengang from 2003 to 2015 (**C**).

*Austinogebia edulis* (Ngoc-Ho & Chan, 1992) is an important seafood item from the coastal intertidal zone of western Taiwan^14–17^. The locals of western Taiwan catch and consume this mud shrimp extensively and egg-bearing females are the most targeted items of harvesting^14^. Mud shrimps dwell in burrows that can be over a meter in depth^15^. The shells of *A. edulis* are soft, thin, and brown (Fig. 1A). One sampling area for this study is Shengang [Latitude = 24.168094 (°N); Longitude = 120.457894 (°E)], near the Chang-Bin Industrial Park which is situated at the west coast of Taiwan (Fig. 1B). The time series values of the annual average densities (mean ± SD) recorded in the Shengang area during the period from 2003–2015 is shown in Fig. 1C. The highest density was recorded in 2003 (31.772 ± 13.27 individuals m^−2^), followed by 2014 (31.770 ± 1.67 individuals m^−2^), whereas the lowest density was recorded in 2007 (4.18 ± 2.51 individuals m^−2^). Possibly because of the chemical industry in the industrial area, the population of *A. edulis* began to decrease dramatically from 2004 (Fig. 1C). Following this sharp population decline, a protected area of *A. edulis* was established in Shengang, Changhua County in 2005. Our sampling area is outside the protected area for *A. edulis*, where its population density was oscillating from 2005 to 2015. Another sampling area for this study is Wangong [Latitude = 23.968126 (°N); Longitude = 120.323173 (°E)], which is a traditional fishing port where thin mangrove forests are distributed along the coastline (Fig. 1B). It is easier to collect samples at Wangong compared to Shengang. These areas are all located in the main production area of the mud shrimp *A. edulis*.

**Figure 2:**
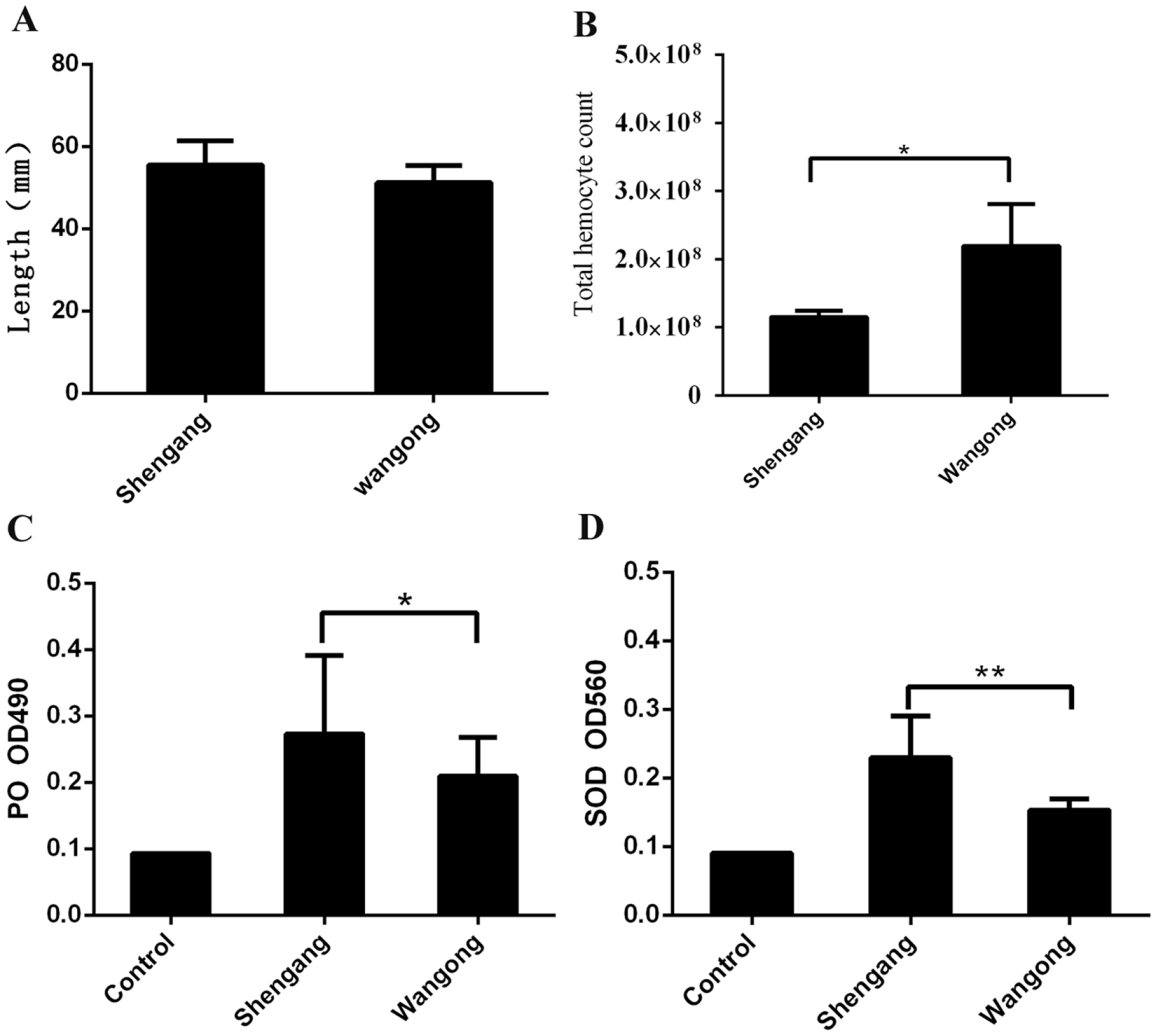
The body length (**A**), total hemocyte count (**B**), PO activity (**C**), and SOD activity (**D**) of *Austinogebia edulis* in Shengang or Wangong. Data are shown as means ± standard deviation (SD) of three separate individuals in the tissues. Means in the same column sharing the same superscript letter are not significantly different and determined by Tukey’s test (*p* > 0.05). Double asterisks indicate a highly significant difference (*p* < 0.01), and single asterisks indicate a significant difference (*p* < 0.05). The control in C and D indicates that there is no hemolymph in the test sample.

No significant difference was found in the body length of *A. edulis* between Wangong and Shengang (Fig. 2A). The average values of the two groups are over 5 cm, which is a normal length of adult *A. edulis* according to an earlier study^14^. Innate immunity defends against microbial infections among crustaceans. It consists of the following mechanisms: antibiotic peptides, melanization, coagulation, encapsulation, and phagocytosis^18^. According to previous studies, cell-mediated immunity is very important for shrimps to survive in the aquatic environment^19^. The total hemocyte counts showed a significantly higher value in Wangong compared to Shengang (Fig. 2B). Total hemocyte counts of the marine invertebrate ascidian *Styela plicata* were explained in an earlier study by organic mercury pollution^20^. This would also explain a lower resistance of *A. edulis* to the external environment and pathogens in Shengang. The WSSV infection can induce high peroxidase (PO) and sodium dismutase (SOD) activity in shrimp^21^. At the same rate of WSSV infection, environmental pollution stress may result in higher immunological parameters, such as up-regulated PO and SOD activity in Shengang (Fig. 2C,D). Water environmental stress may boost the phenoloxidase system of the mud shrimps and increase the PO activity as a protective response^22^. Some studies showed that the SOD activity of aquatic animals was significantly higher in polluted areas than that of animals in less polluted areas^23,24^. However, when the stress response of shrimp reaches certain threshold levels or shrimp are under stress for extended times, this will lead to metabolic disorders in shrimps.

**Figure 3:**
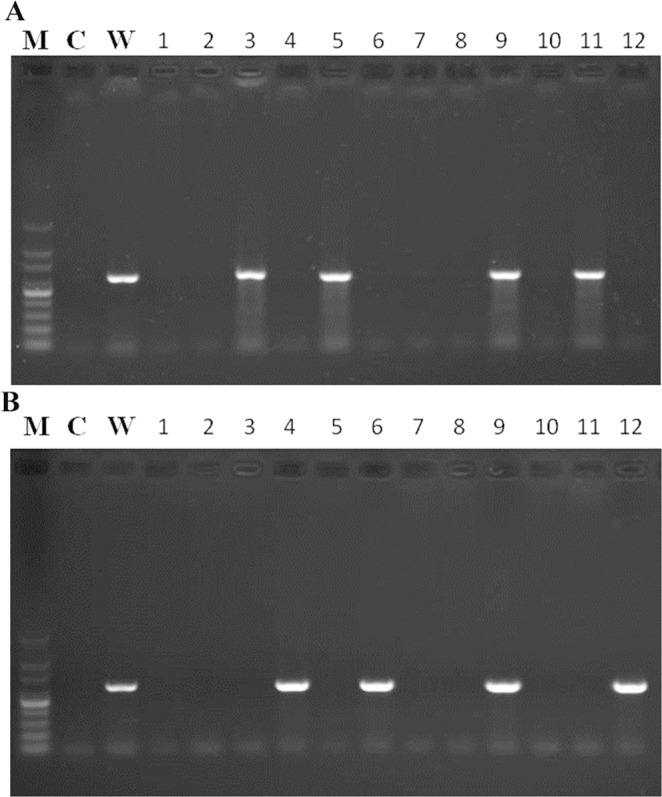
WSSV VP28 DNA detection by PCR in hemocytes of *Austinogebia edulis* in Shengang (**A**) and in Wangong (**B**). M indicates the DNA marker (100 bp–1000 bp); C indicates the negative control; W indicates the positive control (WSSV).

The PCR detection results demonstrated that WSSV was present in hemocytes of *A. edulis* in Shengang and in Wangong (Fig. 3). The same rate of WSSV infection (12/36, 33.33%) was found in Shengang and Wangong. After WSSV infection, environmental pollution stress may have caused a higher mortality in *A. edulis* than in the control. Thus, the population size of *A. edulis* showed a sharp decline in Shengang, whereas it was stable in Wangong (data not shown). This came along with a strong correlation of WSSV infection and environmental pollution stress with the innate immunity of the mud shrimp *A. edulis*. The important viral pathogen WSSV is believed to cause substantial losses in the harvest of shrimp cultures worldwide. It can infect several crustaceans such as shrimp, crab, crayfish, and lobster^25^. Now it became a latent virus in crustaceans that causes sublethal effects in unaffected environments. However, its presence will reduce the innate immunity of crustaceans, and other stressors are expected to do the same. This way sublethal or lethal effects will increase.

**Figure 4:**
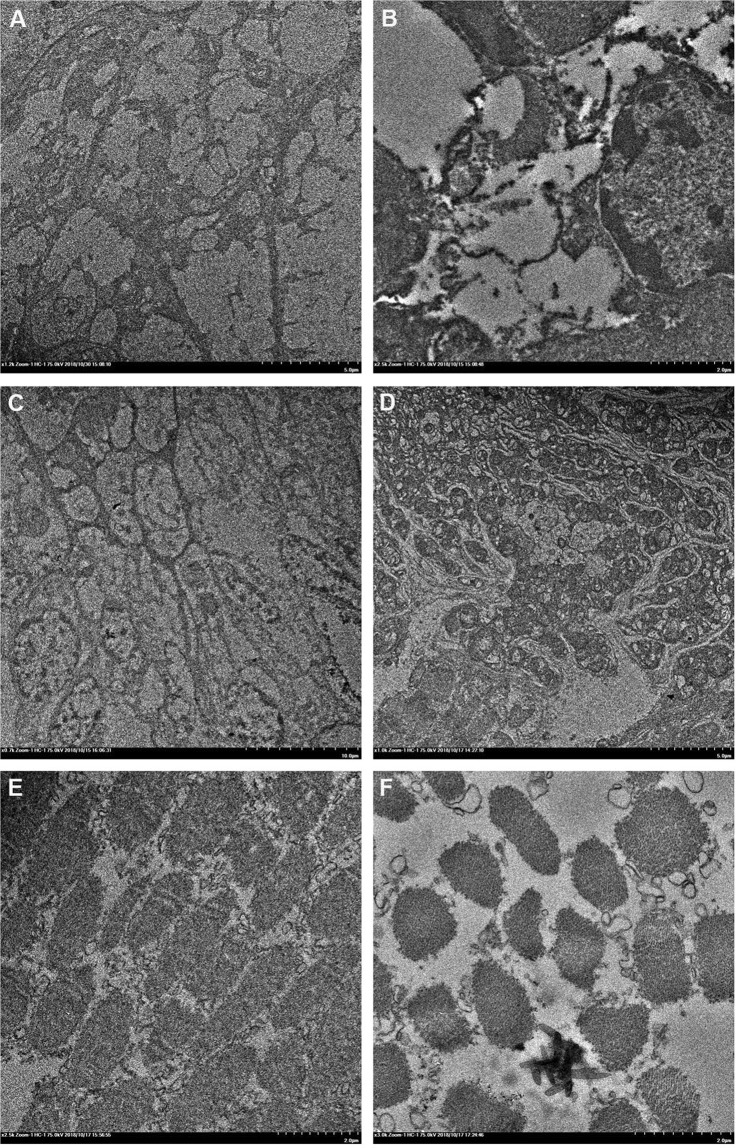
Transmission electron micrograph of major organs in *Austinogebia edulis*. Gill (**A**, non-infected individual, scale bar = 5 µm; **B**, WSSV-infected individual, scale bar = 2 µm); hepatopancreas (**C**, non-infected individual, scale bar = 10 µm; **D**, WSSV-infected individual, scale bar = 5 µm); muscular organ (**E**, non-infected individual, scale bar = 2 µm; **F**, WSSV-infected individual, scale bar = 2 µm).

Transmission electron microscopy showed obvious histological differences in the cell morphology of gills, hepatopancreas, and muscle in WSSV-infected *A. edulis* compared with non-infected *A. edulis* (Fig. 4). Compared to non-infected *A. edulis*, the cell morphology of gills showed cell vacuolization and bursting in WSSV-infected *A. edulis* (Fig. 4A,B). Compared to non-infected *A. edulis*, the cell morphology of the hepatopancreas showed a chaotic arrangement and cell gaps in WSSV-infected *A. edulis* (Fig. 4C,D). Compared to non-infected individuals, the cell morphology of muscle showed larger cell gaps in WSSV-infected *A. edulis* (Fig. 4E,F). The observation that WSSV are likely to break out upon environmental challenges suggests that shrimps contain this virus in a less virulent or dormant state^9,26^. Changes in either the presence, virulence, or dormant state of WSSV may also cause the observed *A. edulis* population oscillations that are indicated in Fig. 1C. Infection brings abnormal changes to the tissues of mud shrimps, so that a latent infectious condition will not kill shrimps immediately^27^. However, latent WSSV infections will reduce the resistance of shrimps to a challenging environment, and make them more susceptible to sublethal xenobiotic contamination.

This study is the first report of WSSV infection in the mud shrimp *Austinogebia edulis*. We hypothesize that a population decrease of *A. edulis* in Shengang is related to WSSV infection. However, at the same rate of WSSV infection, a major factor inducing a population decline can be environmental pollution which also is supposed to be responsible for a massive reduction of hemocytes and an abnormal increase of PO and SOD activity. To conclude, WSSV infection might not be the only reason for the observed oscillations of population densitiesof *A. edulis* in Shengang. The observed population oscillations are likely also associated with stress from environmental pollution as emphasized by Middelboe & Brussaard in 2017^28^. As there is still no effective treatment of WSSV by antibiotics nor vaccines, improving the water and sediment quality of the coastal environment remains the most effective way to protect the populations of this feral shrimp species.

## Methods

### Sampling strategy

Following Das *et al*.^16^ we chose two sampling areas from north to south, which are tourist attractions in Changhua County (Fig. 1). The areas of investigation were: Shengang [Latitude = 24.168094 (°N); Longitude = 120.457894 (°E)] in the northern part located close to the industrial park and Wangong [Latitude = 23.968126 (°N); Longitude = 120.323173 (°E)] in the southern part along the western coast of Taiwan facing the Taiwan Strait. The Changhua coastal industrial park was constructed from 1979 to October 1993. It belongs to a comprehensive industrial area, including food, glass, textile, plastic, chemical, metal, electric power, steel, machinery, hardware, wood, gas, resource recycling, hospitals, transportation, warehousing, and car testing centers. Our study was permitted and supported by the Industrial Development Bureau, Ministry of Economic Affairs. The climate of Taiwan is affected by seasonal monsoons with an average air temperature being 12 °C in winter and 35 °C in summer. We conducted field sampling from July to September 2018. Individuals of the mud shrimp *A. edulis* were carefully collected using a shovel. A total of 100 shrimp samples were randomly collected from each area. The mud shrimp samples were transported from the field to the laboratory in an incubator at low temperature conditions (about 4 °C) on the second day; then healthy shrimps were also selected to collect tissues for analysis in the laboratory.

### Polymerase chain reaction study of WSSV

Zhu *et al*.^29^ was followed here. Briefly, total DNA was extracted from thirty-six individuals of sacrificed mud shrimp with an animal organ genomic DNA mini-prep kit (Sangon, Shanghai, China P.R.). The samples were tested with the primer set VP28-FW and VP28-RV (5′-GCGGTCGACAATGGATCTTTCTTTCAC-3′/5′-ATAGGATCCAACTCGGTCTCAGT-3′), amplifying a part of the WSSV VP28 gene which was used to screen for WSSC-positive animals. PCR was performed with the VP28 primer pair using the following protocol: 5 min at 94 °C followed by 35 cycles at 94 °C for 1 min, at 55 °C for 1 min, and at 72 °C for 1 min. The PCR products were analyzed by electrophoresis on 1% agarose gels stained with ethidium bromide and visualized by ultraviolet transillumination.

### Superoxide dismutase (SOD) assay

Biochemical analyses followed Das *et al*.^17^. In brief was superoxide dismutase activity determined according to the previous reports using nitro blue tetrazolium (NBT) chloride in the presence of riboflavin. Briefly, 100 mL of hemolymph was homogenized in a mechanical homogenizer containing 0.5 mL of phosphate buffer (50 mM, pH 7.8). The homogenate was centrifuged for 5 min at 6000 × g at 4 °C and the supernatant was heated for 5 min at 65 °C to obtain another supernatant after centrifugation (crude extract), which was stored at 20 °C until use. Samples were maintained on ice at all times to avoid protein denaturation. A mixture of NBT, 20 mM of reaction mixture (0.1 mM Ethylenediaminetetraacetic acid (EDTA), 13 mM Methionine, 0.75 mM NBT, and 20 mM Riboflavin in phosphate buffer, 50 mM, pH 7.8) and 0–100 mL of crude extract were placed under fluorescent light for 2 min or until A560 in the control tubes reached 0.2 to 0.25 optical density (OD). The results were expressed as relative enzymatic activity.

### Prophenoloxidase (proPO) assay

Prophenoloxidase activity was measured spectrophotometrically by recording the formation of dopachrome produced from L-dihy-droxyphenyl alanine according to the reported method^30^. Briefly, the diluted hemolymph was centrifuged at 800 × g at 4 °C for 20 min to collect a pellet which was resuspended gently in cacodylate buffer (0.01 M sodium cacodylate, 0.45 M sodium chloride (1.10 M Trisodium citrate, pH 7.0). The suspended pellet was centrifuged again and the pellet was re-suspended with 100 mL of cacodylate buffer. The re-suspended pellet was incubated with 50 mL trypsin (T-0303, Sigma, 1 mg/mL) at 25 °C for 10 min, which served as an activator; 50 mL L-DOPA was then added, following the addition of 800 mL of cacodylate buffer 5 min later. The optical density at 490 nm was measured using spectrophotometer-117 (Systronics, Shanghai, China).

### Total hemocyte count (THC) assay

Following the method of Sun and coworkers^30^, hemolymph (100 mL) was withdrawn from the ventral sinus of each mud shrimp into a 1 mL sterile syringe (25 gauge) containing 0.9 mL anticoagulant solution (Trisodium citrate 30 mM, sodium chloride 0.34 M, EDTA 10 mM, pH 7.55). A drop of the anticoagulant-hemolymph mixture (100 mL) was placed on a hemocytometer, and a THC was made under an inverted phase-contrast microscope (Leica DMIL, Leipzig, Germany)^31^.

### Ultrastructural studies

Animals used for transmission electron microscopy (TEM) were dissected in the field and the tissues were fixed in 4 °C 2.5% glutaraldehyde following Rodriguez^6^. Tissues were postfixed in 1% osmium tetroxide (OsO_4_) in 100 mM sodium cacodylate buffer for 1 h, further processed and embedded in Spurr’s (TM) resin. Sections were cut by a Reichert Ultracut OMU3 microtome (Leica, Leipzig, Germany) at 100 nm thickness, stained with uranyl acetate/70% methanol and lead citrate and examined with a Hitachi HT 7700 transmission electron microscope (Hitachi, Tokyo, Japan).

### Data analysis

We followed Ma and co-workers^9^ here by obtaining the mean and standard deviation of three repeated experiments by one-way ANOVA with three independent experimental data. Unidirectional variance analysis was used to estimate statistical differences in accordance with the lowest bit difference and Duncan multiple comparisons. All measurements were analyzed using SPSS 19. A probability level 0.01 was used to show statistically significant differences (*p* < 0.01).

### Ethical approval and informed consent

The sampling of mud shrimps was approved and supported by the Industrial Development Bureau, Ministry of Economic Affairs.
